# Controllable two-scale network architecture and enhanced mechanical properties of (Ti_5_Si_3_+TiBw)/Ti6Al4V composites

**DOI:** 10.1038/srep32991

**Published:** 2016-09-13

**Authors:** Y. Jiao, L. J. Huang, T. B. Duan, S. L. Wei, B. Kaveendran, L. Geng

**Affiliations:** 1 School of Materials Science and Engineering, Harbin Institute of Technology, P.O. Box 433, Harbin 150001, P. R. China; 2 State Key Laboratory of Advanced Welding and Joining, Harbin Institute of Technology, P.O. Box 433, Harbin 150001, P. R. China

## Abstract

Novel Ti6Al4V alloy matrix composites with a controllable two-scale network architecture were successfully fabricated by reaction hot pressing (RHP). TiB whiskers (TiBw) were *in-situ* synthesized around the Ti6Al4V matrix particles, and formed the first-scale network structure (FSNS). Ti_5_Si_3_ needles (Ti_5_Si_3_) precipitated in the β phase around the equiaxed α phase, and formed the secondary-scale network structure (SSNS). This resulted in increased deformation compatibility accompanied with enhanced mechanical properties. Apart from the reinforcement distribution and the volume fraction, the ratio between Ti_5_Si_3_ and TiBw fraction were controlled. The prepared (Ti_5_Si_3_ + TiBw)/Ti6Al4V composites showed higher tensile strength and ductility than the composites with a one-scale microstructure, and superior wear resistance over the Ti6Al4V alloy under dry sliding wear conditions at room temperature.

Recent studies reveal that titanium matrix composites (TMCs); especially, discontinuously reinforced titanium matrix composites (DRTMCs) reinforced with ceramic particles are gaining widespread attention, due to their outstanding mechanical properties. DRTMCs have become promising materials for aerospace, automotive and military applications due to their isotropic properties, high specific strength, high modulus, good wear resistance and high temperature durability^1^,^2^. Among various manufacturing methods of DRTMCs, a combination of reaction hot pressing (RHP) and *in-situ* technology has been widely investigated and developed, because of its near net-shape processing and low cost, especially its ability to make composites obtain superior mechanical properties, clean interface and strong interface bonding^3^.

In considerable amount of literatures studying DRTMCs, *in-situ* TiB whisker (TiBw)^4^,^5^,^6^ and TiC particles (TiCp)^7^,^8^ have been unanimously considered as the best reinforcements, due to their desirable properties, such as high strength, modulus, good chemical stability and similar coefficient of thermal expansion (CTE) with Ti matrix. In addition, hybrid reinforcements, such as TiBw and TiCp^9^, TiBw and La_2_O_3_^10^ were extensively utilized to pursue superior mechanical properties. However, Ti_5_Si_3_ particles have rarely been used as reinforcement during the fabrication of TMCs via *in-situ* methods. Ti_5_Si_3_ phase possesses many excellent properties, such as high melting point (2130 °C), low density (4.26 g/cm^3^), retaining strength up to 1200 °C, especially good oxidation and creep resistance at elevated temperatures^11^. Furthermore, the CTE of Ti_5_Si_3_ is also close to that of Ti alloys^12^. Ti_5_Si_3_ can potentially be regarded as an attractive wear-resistant and corrosion-resistant material due to its inherent high hardness, unique chemical composition and strong covalent bonds^13^. Therefore, Ti alloy matrix composites reinforced with Ti_5_Si_3_ particles are potential candidates for high temperature applications.

Inadvertently, previous investigations have usually pursued DRTMCs with a homogeneous microstructure. Unfortunately, the homogeneous composites have always showed inferior ductility, even extreme brittleness for those fabricated by conventional power metallurgy, except for limited improvement in strength. In recent years, on the basis of Hashin-Shtrikman (H-S) theory, Huang *et al*.^1^,^4^,^5^ designed and fabricated a series of DRTMCs with a quasi-continuous network structure of reinforcements using low-energy milling and RHP processes combined with *in-situ* technology. Sufficient results illustrate that the network architecture is beneficial to inspire strengthening effect of reinforcement and toughening effect of matrix alloy in DRTMCs. However, the large network structure limits reinforcement fraction, which restricted further improvement in the strength of composites.

Microstructural architectures with material specific design have proven to be powerful in nature and engineering applications. Natural materials, such as shells^14^, tooth^15^, and bone^16^, exhibit multi-scale hierarchical structures spanning from microscopic to macroscopic length scales and show significantly improved mechanical properties (e.g. strength and toughness) compared to base materials. For this reason, there are increasing efforts to artificially create or imitate natural materials’ structures in composites.

In this study, not only the quasi-continuous network architecture and the best TiBw reinforcement were adopted, but also low-cost micro Si particles were used to achieve Ti_5_Si_3_ reinforcement in the novel lower secondary-scale structure by precipitation, in order to further improve the mechanical properties of the composites. Ti6Al4V alloy as a typical one of dual-phase titanium alloys was selected as the matrix due to its superior mechanical properties and extensive usage. Therefore, in the present work, the aim is to tailor the distribution of Ti_5_Si_3_ and TiBw reinforcements, so as to create a controllable two-scale network structure and enhance the mechanical properties of titanium alloy matrix composites.

## Results and Discussion

### Microstructure

**Figure 1:**
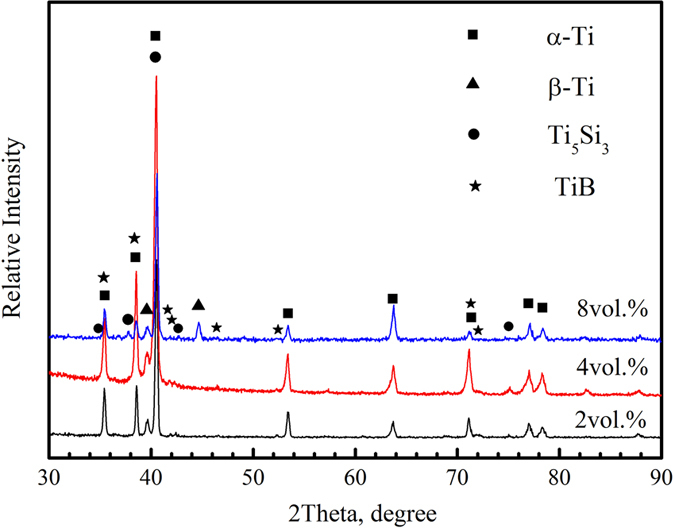
X-ray diffraction patterns of the fabricated composites with different volume fractions of Ti_5_Si_3_ reinforcement.

Figure 1 shows the X-ray diffraction patterns of the as-sintered composites with different Ti_5_Si_3_ volume fractions fabricated at 1300 °C for 1.5 h. It presents that the as-sintered composites mainly consist of Ti, Ti_5_Si_3_ and TiB phases. Moreover, no Si and TiB_2_ phases were detected. This result demonstrates that the *in-situ* reaction between Ti and TiB_2_ was likely to react completely according to the reaction in Eq. (1). Ti_5_Si_3_ particles precipitated during the cooling process, due to the decreased solubility of Si element in the β phase. It is evident from Fig. 1 that there are no significant differences in the XRD patterns of the composites with different Ti_5_Si_3_ fractions except for the difference in the peak intensities.

**Figure 2:**
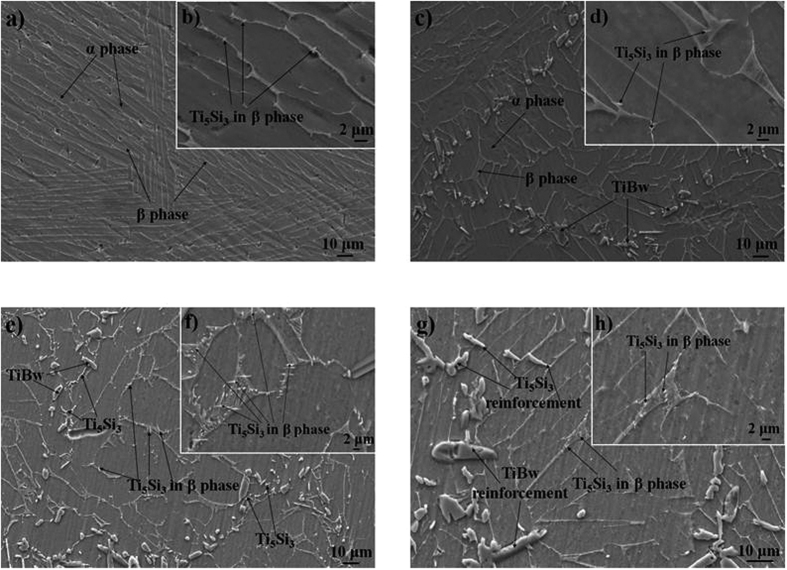
SEM micrographs of (**a**,**b**) 4vol.%Ti_5_Si_3_/Ti6Al4V, (**c**,**d**) (2vol.%Ti_5_Si_3_ + 3.4vol.%TiBw)/Ti6Al4V, (**e**,**f**) (4vol.%Ti_5_Si_3_ + 3.4vol.%TiBw)/Ti6Al4V and (**g**,**h**) (8vol.%Ti_5_Si_3_ + 3.4vol.%TiBw)/Ti6Al4V; (**a**,**c**,**e**,**g**) at relatively low magnification and (**b**,**d**,**f**,**h**) at relatively high magnification.

Figure 2a shows SEM micrograph of the 4vol.%Ti_5_Si_3_/Ti6Al4V composite. It can be seen from Fig. 2a that the microstructure of the 4vol.%Ti_5_Si_3_/Ti6Al4V composite is similar to that of the Ti6Al4V alloy, due to the absence of the first-scaled TiBw/Ti6Al4V network structure. The observed microstructure is a typical α + β lamellar structure, which belongs to the widmanstätten microstructure. When α + β two-phase Ti alloys are cooled slowly (furnace cooling) from a temperature above the β transus, the widmanstätten microstructure is usually obtained^4^. In addition, β grains with large size of about 1 mm replace Ti6Al4V particle with 150 μm, which indicates that the Ti6Al4V particles entirely merged, while Si particles entirely solid soluted into the β phase. During sintering process, the Si element totally diffused into the β phase. Figure 2b shows needle-like Ti_5_Si_3_ precipitated and distributed in the β phase, which was around the α phase. This phenomenon can be interpreted as: during furnace cooling, the solubility of Si element in the β-Ti decreases with decreasing temperature; the fraction of β phase also decreases due to the phase transition (β → α).

Figure 2c–h show SEM micrographs of the (Ti_5_Si_3_ + TiBw)/Ti6Al4V composites with different fractions of Ti_5_Si_3_ reinforcement. Only Ti, Ti_5_Si_3_ and TiB phases were observed, which is consistent with the XRD result (Fig. 1). It can be clearly seen from Fig. 2c that the TiBw reinforcement distributed around the Ti6Al4V particles, and formed an analogous “grain boundary” structure. The “grain” size is about 150 μm equal to that of the Ti6Al4V raw material. On the whole, the TiBw reinforcement formed the regular first-scale network structure (FSNS, Fig. 2c), as reported in the TiBw/Ti6Al4V composite^4^. On the lower scale, few Ti_5_Si_3_ particles precipitated in the β-Ti phase, which distributed around the α-Ti phase, and formed the secondary-scale network structure (SSNS, Fig. 2d), due to the first-scale network structure. Since the two reinforcements distributed in different scales, these two-scale networked composites are different from the conventional hybrid-reinforced composites with homogeneous dispersion of two reinforcements^9^,^10^. The lower SSNS of Ti_5_Si_3_ reinforcement can strengthen the interior matrix particles effectively, due to the precipitation of fine Ti_5_Si_3_ needles^17^. Moreover, the SSNS can increase deformation compatibility, which is beneficial to the strength and ductility of composites. Previous results^18^ have indicated that the precipitation of S1 (silicide) leads to an increase in the strength of (TiB + La_2_O_3_)/Ti composites. When the fraction of Ti_5_Si_3_ reinforcement is increased to 4vol.%, as shown in Fig. 2f, more needle-like Ti_5_Si_3_ particles precipitated in the β-Ti phase compared with that in Fig. 2d. Figure 2e,f show some fine and needle-like Ti_5_Si_3_ particles formed in the β-Ti phase. The possible reasons for this phenomenon are as follows. According to the Ti-Si phase diagram^19^, the solubility of Si in β-Ti phase is around 3 wt.% at 1300 °C, while it nearly does not solid solute in α-Ti at room temperature. Therefore, the entire Si can dissolve into β-Ti phase at 1300 °C. However, when cooling from 1300 °C, the volume fraction of β-Ti phase decreases due to phase transformation from β-Ti to α-Ti. The saturation of Si element leads to the precipitation of Ti_5_Si_3_ phase in the β-Ti phase. It is worth pointing out that Si element is considered to work as a β-stabilizer in Ti alloys^20^. Therefore, the volume fraction of β-Ti can be slightly increased, which may prove to be beneficial to the ductility of composites. The needle-like Ti_5_Si_3_ reinforcement distributed in the β-Ti phase around the near equiaxed α-Ti phase, and formed the secondary-scale network structure (SSNS). Gu *et al*.^21^ studied the mechanical alloying of Ti-Si powder mixture using high-energy ball milling at ambient temperature. A significant increase in solid solubility of Si in Ti was achieved by mechanical alloying. The shrinkage of Ti lattice was caused by diffusion of Si atoms into Ti. In a previous literature^22^, La atoms would solid solute into β-Ti firstly, followed by their precipitation from β-Ti during the phase transformation from β-Ti to α-Ti. Following which the La atoms reacted with oxygen inside the matrix alloy, forming fine and homogeneously distributed La_2_O_3_ particles on the grain boundary of the titanium alloy matrix.

Noticeably, comparing Fig. 2a with Fig. 2e, a remarkable difference in the Ti_5_Si_3_ reinforcement was observed between the monolithically reinforced composite and the composite with a two-scale microstructure. In the (Ti_5_Si_3_ + TiBw)/Ti6Al4V composite, a small quantity of needle-like Ti_5_Si_3_ reinforcements distributed near the TiBw reinforcements. Most of the Ti_5_Si_3_ particles were observed in the β-Ti phase of the SSNS. Li *et al*.^18^ found that few silicides are distributed in the α platelet. Previous results have shown that Nb_5_Si_3_ particles precipitated at the γ/γ interface and the γ/α_2_ interface^23^. However, entire Ti_5_Si_3_ particles precipitated in the β-Ti phase in the Ti_5_Si_3_/Ti6Al4V composite. Owing to the absence of distribution of the TiBw reinforcement in the first-scale network region (FSNR), the formation of the FSNS did not take place.

Besides the central β phase of the Ti6Al4V particles, few Ti_5_Si_3_ particles were observed around the TiBw reinforcements (Fig. 2e). This phenomenon may be due to the addition of TiBw, which increased the amount of defects. Sun *et al*.^24^ prepared TiAl alloys using non-consumable electrode arc melting in an argon atmosphere. Ti_5_Si_3_ particles preferred to precipitate at stacking faults. The growth of needle-like Ti_5_Si_3_ particles at the γ/γ boundaries was controlled by interfacial diffusion of Si, with an incoherent γ/Ti_5_Si_3_ interface. In addition, the Ti_5_Si_3_ phase distributed around the TiBw reinforcement with a size of around 1 μm; whereas, the size of the Ti_5_Si_3_ precipitation in the β-Ti phase is about 400 nm. Si element prefers to diffuse to areas with defects and precipitate during cooling process.

However, when the fractions of Ti_5_Si_3_ reinforcement is as high as 8vol.%, as shown in Fig. 2h, lesser Ti_5_Si_3_ particles precipitated in the β-Ti phase of the central Ti6Al4V particles as compared to that in Fig. 2f. In contrast, coarse Ti_5_Si_3_ particles formed in the vicinity of TiBw reinforcements. This may be attributed to the limited solubility of Si in the β-Ti phase. Redundant Si reacted with Ti to *in-situ* synthesize Ti_5_Si_3_ particles. This increased the local volume fraction of ceramic phases in the FSNR, whose energy was increased. Therefore, Si element in the β-Ti phase prefers to nucleate and grow in the FSNR during cooling process. The connected and coarse Ti_5_Si_3_ particles are certainly harmful to the properties of the composites^25^.

According to the Ti-Si phase diagram^19^, the silicide in the equilibrium microstructure of Ti-Si should be Ti_3_Si. However, there are many contradictions in previous literatures, about the most thermodynamically favorable structure of precipitation at low concentrations of Si^26^,^27^,^28^. Poletaev *et al*.^26^ applied a recently developed thermodynamic model to predict the structure of Ti-Si precipitations in the α-Ti matrix. They discovered that formation of the Ti_5_Si_3_ phase is more favorable than that of the Ti_3_Si phase in contradiction. The theoretical framework was confirmed with experimental investigations of microstructure.

**Figure 3:**
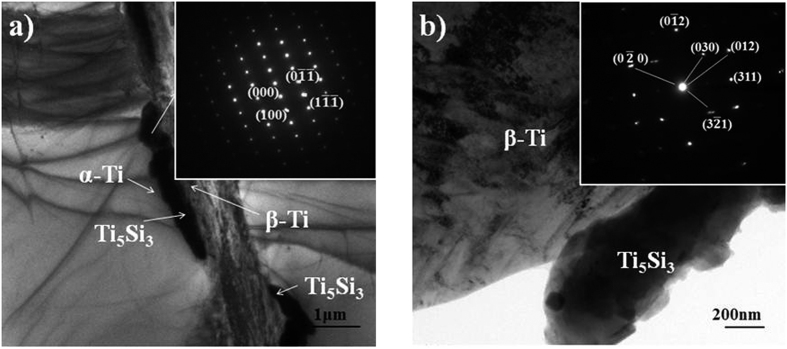
TEM image and selected area diffraction (SAD) patterns of the Ti_5_Si_3_ particle (**a**), corresponding (SAD) pattern (**b**) on the Ti_5_Si_3_/β-Ti interface in the (4vol.%Ti_5_Si_3_ + 3.4vol.%TiBw)/Ti6Al4V composite.

To further determine the crystal structure of the precipitated particles, TEM analysis was conducted. The corresponding morphologies and selected area diffraction (SAD) patterns are shown in Fig. 3. Figure 3a further confirmed that the precipitations are Ti_5_Si_3_ phase rather than Ti_3_Si phase. Ti_5_Si_3_ phase is a common precipitation in Si-containing Ti alloys. SAD analysis in Fig. 3b found that the Ti_5_Si_3_ particle has certain crystal relationships with the matrix β-Ti, which can be presented as:


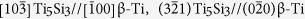


From the TEM image in Fig. 3a it is evident that the Ti_5_Si_3_ phase precipitated at the α/β interface. This result is consistent with the SEM observations. This is also in agreement with the previous results showing that very fine silicides were formed at α/β interfaces^23^.

**Figure 4:**
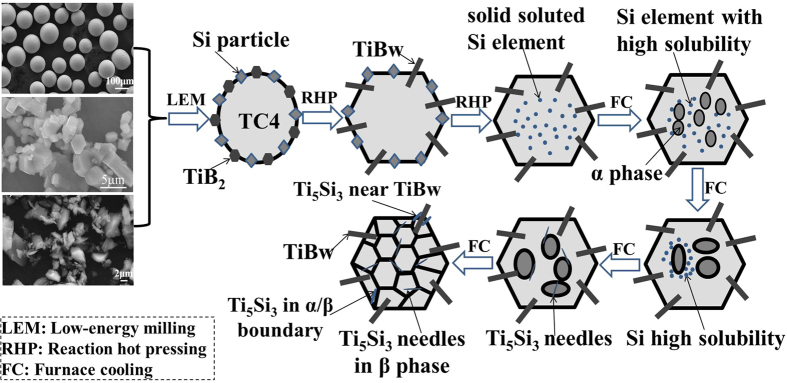
Schematic illustrations of the formation mechanism of Ti_5_Si_3_ needles and two-scale network distribution.

Combing with the above analysis, the formation and distribution mechanisms of secondary-scale Ti_5_Si_3_ phase can be illustrated in Fig. 4. During sintering process, Si completely diffused into the Ti6Al4V matrix from the Ti6Al4V particle surface. During the following cooling process, a portion of Si element distributed around the precipitated α-Ti phase and TiB whisker, a large proportion of Si element distributed in the residual β-Ti phase. Ti_5_Si_3_ needles precipitated with the decrease in temperature.

**Figure 5:**
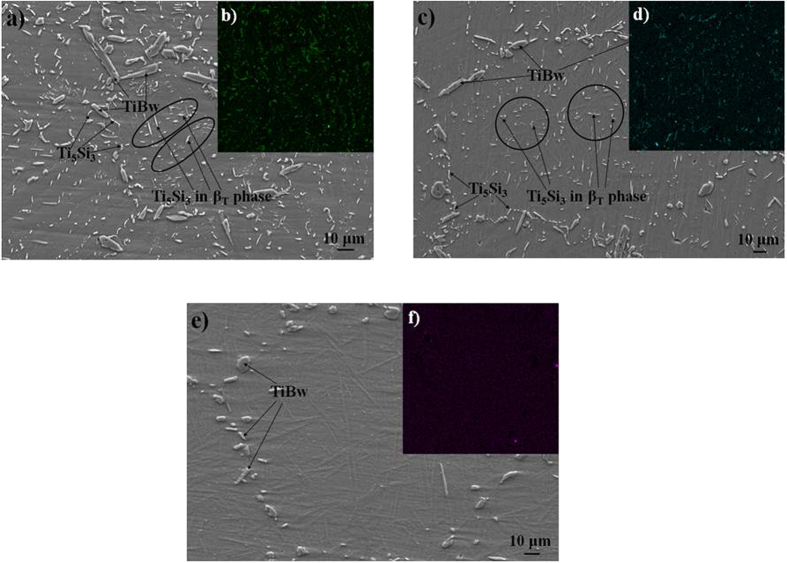
SEM micrographs and Si element distribution of (4vol.%Ti_5_Si_3_ + 3.4vol%TiBw)/Ti6Al4V composites after solid solution treatment at (**a**) 990 °C, (**b**) 1100 °C, (**c**) 1200 °C for 40 min followed by WQ.

In order to adjust the precipitation sites of the Ti_5_Si_3_ phase, (4vol.%Ti_5_Si_3_ + 3.4vol.%TiBw)/Ti6Al4V composites were heat treated at 990 °C, 1100 °C and 1200 °C for 40 min. SEM micrographs of the heat treated composites are shown in Fig. 5. After heat treatment, there is no change in the microstructure and distribution of the TiBw reinforcement, whereas there is a clear change in the microstructure of the Ti6Al4V matrix and Ti_5_Si_3_ reinforcement. The bright β phase in the Ti6Al4V matrix is converted into the transformed β microstructure (β_T_), which consists of the residual β phase, martensite α’ phase formed in the WQ process. Furthermore, the contrast between the primary α phase and the transformed β microstructure decreases with increasing quenching temperatures, which demonstrates that the increasing fraction of the martensite and the decreasing fraction of β phase in β_T_. The increased fraction of the martensite is beneficial to the hardness and strength of composites^29^. Comparing Fig. 5 with Fig. 2, the volume fraction of β_T_ in the heat treated composite is much higher than that of the stable β phase in the as-sintered composite. Figure 5 also indicates that the volume fraction of β_T_ increases with increasing WQ temperatures. While, the fraction of Ti_5_Si_3_ reinforcement distributed in the vicinity of TiBw reinforcement decreased with increasing quenching temperatures. When the heat treatment temperature reached 1200 °C, Ti_5_Si_3_ phase disappeared near TiBw reinforcement and in the SSNS. The size of the Ti_5_Si_3_ precipitation in the as-sintered composites is around 400 nm, while some Ti_5_Si_3_ precipitation in the heat treated composites even increased to 2 μm or decreased to 0. Therefore, heat treatment can control the size of Ti_5_Si_3_ precipitation. Li *et al*.^18^ reported that both particle size and volume fraction of silicides increase with increasing thermal exposure temperature.

To detect the distribution of Si in the composites, EDX (element surface scanning) analysis was carried out, as shown in Fig. 5. According to the results of element surface scanning, the fraction of Si element in the vicinity of TiBw reinforcement decreased with increasing WQ temperatures. A small quantity of the Ti_5_Si_3_ phase grew and much of the Si element dissolved into the β_T_ when the heat treatment temperature was 990 °C. When the temperature was increased to 1100 °C, more Ti_5_Si_3_ phase became fine and acicular. When the temperature reached 1200 °C, Si was not detected in the vicinity of TiBw reinforcement. This indicates that the Si element entirely dissolved into the β_T_.

### Mechanical properties

**Figure 6:**
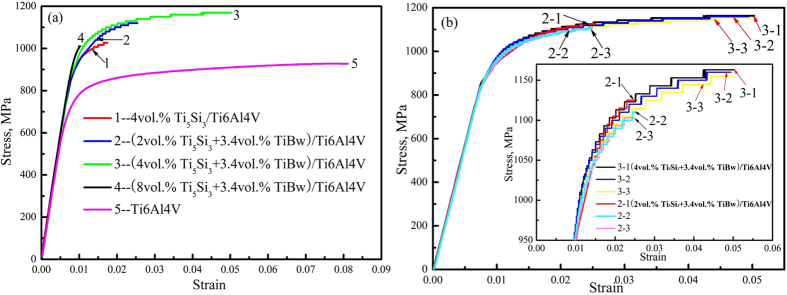
Tensile stress-strain curves of the as-sintered Ti6Al4V alloy and composites at room temperature.

Figure 6a shows the typical tensile stress-strain curves of the as-sintered Ti6Al4V alloy and composites at room temperature. It is obvious that the strength of the (Ti_5_Si_3_ + TiBw)/Ti6Al4V composites significantly increased when compared to those of the monolithically reinforced Ti_5_Si_3_/Ti6Al4V composite and the as-sintered Ti6Al4V alloy. For example, the ultimate tensile strength of the (2vol.%Ti_5_Si_3_ + 3.4vol.%TiBw)/Ti6Al4V composite increased to 1130 MPa from 1030 MPa, when compared to that of the monolithically reinforced Ti_5_Si_3_/Ti6Al4V composite. The yield strength of the (4vol.%Ti_5_Si_3_ + 3.4vol.%TiBw)/Ti6Al4V composite increased to 1004 MPa from 769 MPa, compared with the Ti6Al4V alloy. Moreover, the elongation of the (4vol.%Ti_5_Si_3_ + 3.4vol.%TiBw)/Ti6Al4V composite was retained at 4%, which is much higher than that of the Ti_5_Si_3_/Ti6Al4V composite. This phenomenon can be attributed to the two-scale network microstructure with high deformation compatibility. When compared to the STA Ti6Al4V alloy^30^, the strength of the composites with a two-scale network decreased significantly. However, the elongations show a slight difference. In addition, it is clear that the strength and elongation of the composites increased with increasing volume fraction of Ti_5_Si_3_ from 2vol.% to 4vol.%. The increased elongation is attributed to more Ti_5_Si_3_ precipitations with nano-size in the β-Ti phase, which increase the deformation compatibility of the composites. The deformation compatibility of network unit was improved, and the moving dislocations can bypass the particles instead of shearing them, and then the elongation to fracture can be improved. However, the strength and elongation of the (8vol.%Ti_5_Si_3_ + 3.4vol.%TiBw)/Ti6Al4V composite were inferior over the other composites. The composite did not undergo any plastic deformation, resulting in brittle fracture. This is consistent with the high local volume fraction of reinforcement in the FSNS and the coarse Ti_5_Si_3_ particles in the FSNR. In the fabrication of metal matrix composites, reproducibility is a significant concern. Figure 6b shows reproducibility of the (Ti_5_Si_3_ + 3.4vol.%TiBw) composites with different Ti_5_Si_3_ fractions. In the curves, 3-1, 3-2, 3-3 represents three similar tensile curves of the (4vol.%Ti_5_Si_3_ + 3.4vol.%TiBw) composites, respectively. From the tensile curves, it is obvious that the tensile properties of the composites with a two-scale network structure are reproducible.

In order to further investigate the contribution of the two-scale network distribution to the tensile properties, the (4vol.%Ti_5_Si_3_ + 3.4vol.%TiBw)/Ti6Al4V composites were compared with the (TiBw + TiCp)/Ti6Al4V and TiBw/Ti6Al4V composites with a one-scale network structure. Firstly, compared with the monolithically reinforced 5vol.%TiBw/Ti6Al4V composite^4^, the ultimate tensile strength and yield strength of the (4vol.%Ti_5_Si_3_ + 3.4vol.%TiBw)/Ti6Al4V composite increased by 70 MPa and 64 MPa. Moreover, the elongation increased from 3.6% to 4%. Secondly, in the (4vol.%Ti_5_Si_3_ + 3.4vol.%TiBw)/Ti6Al4V composite, the tensile strength and elongation increased from 1129MPa and 2.4% to 1160MPa and 4%, as compared to the hybrid-reinforced 3vol.%(TiBw + TiC)/Ti6Al4V composite^9^. All the results show that the tensile properties of the (Ti_5_Si_3_ + TiBw)/Ti6Al4V composite with a two-scale network architecture are superior over those of the Ti_5_Si_3_/Ti6Al4V, TiBw/Ti6Al4V and hybrid-reinforced (TiBw + TiCp)/Ti6Al4V composites with a one-scale structure. When compared to the composites fabricated by casting^2^, the present strengthening effect can be viewed as a superior strengthening effect, because not only the strength but also the elongation was improved. This may be attributed to the combination of the FSNS and SSNS. The fine Ti_5_Si_3_ needles in the β-Ti phase within the Ti6Al4V matrix can strengthen the softer β phase and provide dispersion hardening by acting as a barrier for the dislocation movement. Moreover, TiBw distributed in the FSNR strengthened the grain boundary of the composites. Additionally, the formed fine SSNS can effectively increase deformation compatibility and prohibit necking during tensile deformation, resulting in elongation improvement.

Table 1 shows the tensile properties of the as-sintered and heat treated (4vol.%Ti_5_Si_3_ + 3.4vol.%TiBw)/Ti6Al4V composites tested at room temperature, in order to further demonstrate the effect of heat treatment to adjust the distribution of Ti_5_Si_3_ precipitation. With increasing quenching temperatures, the tensile strength of the heat treated composites increased. However, compared with that of the as-sintered composites, the tensile strength after water quenching decreased significantly. Moreover, in those composites, brittle fracture took place immediately. The decreased strength and brittleness may be due to the excessive martensite or hardness, which is generated by the high quenching temperatures^31^.

**Figure 7:**
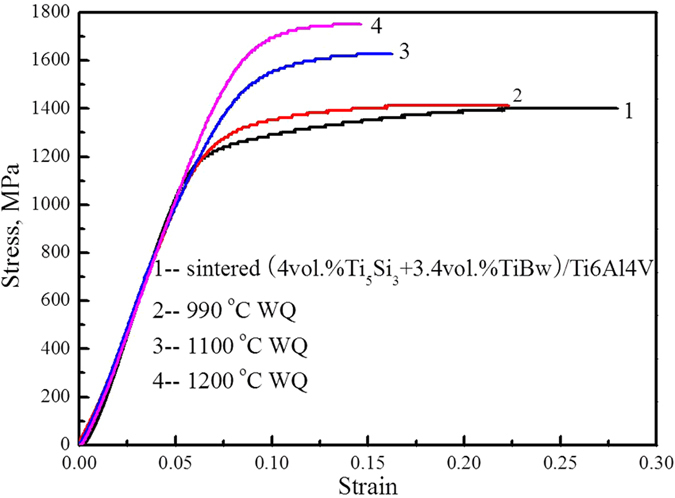
Compressive stress-strain curves of the (4vol.%Ti_5_Si_3_ + 3.4vol.%TiBw)/Ti6Al4V composites before and after heat treatments at room temperature.

In order to evaluate the evolution of properties, compressive tests were performed on the heat treated composites which showed brittle fracture under tensile test conditions. Figure 7 shows the variations in the compressive strength of the composites after water quenching. Furthermore, the compressive properties including yield compressive strength (YCS), ultimate compressive strength (UCS) and fracture strain (ε) of the as-sintered and heat treated (4vol.%Ti_5_Si_3_ + 3.4vol.%TiBw)/Ti6Al4V composites tested at room temperature are shown in Table 2. It can be concluded that the *in-situ* synthesis of TiBw reinforcement and precipitation of Ti_5_Si_3_ phase significantly increased the strength of the as-sintered composite, while decreasing the plasticity correspondingly. The YCS and UCS of the heat treated composites increased with increasing quenching temperatures. The YCS and UCS can be increased to 1687 MPa and 1753 MPa from 1225 MPa and 1402 MPa, respectively. The fraction of the β_T_ in the matrix and the saturation of Si element increased with increasing quenching temperatures, which led to a significant improvement in strength. The other reason is the presence of fine Ti_5_Si_3_ precipitation in the β_T_. Li *et al*.^31^ reported that the influence of heat treatment played an important role in enhancing the strength of the (TiB + La_2_O_3_)/Ti composites. After thermal exposure, the strength of the heat treated specimens increased due to the precipitation of Ti_3_Al and silicides. When compared to the monolithically reinforced 5vol.%TiBw/Ti6Al4V and 5vol.%TiCp/Ti6Al4V composites, the yield strength of the (4vol.%Ti_5_Si_3_ + 3.4vol.%TiBw)/Ti6Al4V composite increased by 28.9%^32^ and 44.1%^33^, respectively. The first-scale TiBw reinforcement and the secondary-scale Ti_5_Si_3_ particles distributed in different regions. The increased reinforcement fractions enhanced the composites strength. Additionally, fine Ti_5_Si_3_ particles can bear higher stress than TiB whiskers. Consequently, the strengthening mechanisms of the composites may be mainly attributed to the “grain boundary” strengthening from first-scale networked TiBw reinforcement and dispersion strengthening from the secondary-scale networked Ti_5_Si_3_ reinforcement. Furthermore, according to the literatures^34^,^35^ and our experience, the composites with a two-scale network structure display superior mechanical properties and oxidation resistance at high temperatures.

Ti alloys are unlikely to have adequate wear resistance for using as structural materials without surface treatment. To overcome the poor wear resistance of Ti alloys, particulate-reinforced TMCs are a suitable solution. A previous study showed that the (TiB + TiC)/Ti composite displays improvement in wear resistance^36^.

**Figure 8:**
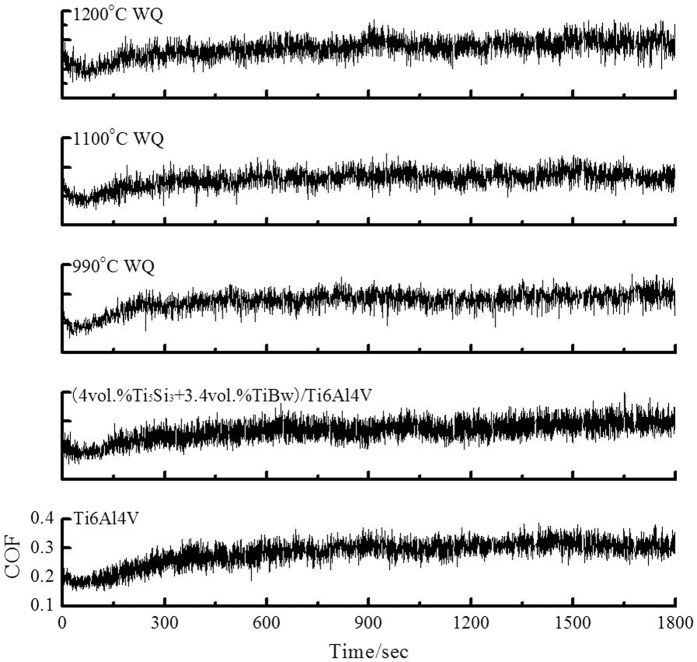
Friction coefficient of the Ti6Al4V alloy, the as-sintered and heat treated (4vol.%Ti_5_Si_3_ + 3.4vol.%TiBw)/Ti6Al4V composites.

The coefficients of friction (COFs) of the Ti6Al4V alloy, the as-sintered and heat treated (4vol.%Ti_5_Si_3_ + 3.4vol.%TiBw)/Ti6Al4V composites under contact load of 10N are shown in Fig. 8. The average COFs and mass loss of the samples are listed in Table 3. It can be seen from Fig. 8 that all the samples showed a shorter and unstable friction period (about 300s) in the initial stage of friction tests. The COF underwent an initial fluctuation followed by a steady tendency. When compared to the as-sintered Ti6Al4V alloy, the average COF of the as-sintered (4vol.%Ti_5_Si_3_ + 3.4vol.%TiBw)/Ti6Al4V composite decreased by 7.0%. This suggests the addition of reinforcements resulted in a change in wear mechanism and a decrease in COF compared with the unreinforced matrix alloy. On one hand, the strength and hardness of two-scale network structured composites are higher than the Ti6Al4V alloy. Especially, the finer Ti_5_Si_3_ particles improved the hardness and strength of the softer β-Ti matrix. The adhesive effect of the composites against their counterparts was limited and the proportion of asperities was reduced during friction process. On the other hand, the increased flash temperature resulted in the softening of the Ti6Al4V matrix. However, the introduction of the reinforcements improved the high temperature strength of the composites. Therefore, the scuffing effect of the composites against their counterparts was alleviated. The mass loss of the as-sintered composite is lower than that of the Ti6Al4V alloy. It means that the composite has superior wear resistance over the matrix alloy, due to the reinforcement with high hardness.

It is worth pointing out that the mass loss of the heat treated composites is slightly higher than that of the as-sintered composites. The mass loss of the heat treated composites decreased with increasing quenching temperatures. When the heat treatment temperature was 990 °C, some growing Ti_5_Si_3_ phases resulted in a decrease in wear resistance. When the temperature was increased to 1100 °C, more Ti_5_Si_3_ phases became fine and acicular, which is beneficial to the wear resistance of the composites. At 1200 °C, the excessive martensite resulted in high hardness and Si element entirely dissolved into the β_T_ strengthened the matrix alloy. Therefore, the wear resistance of the composite after water quenching at 1200 °C is superior over the as-sintered composite.

## Materials and Methods

To manufacture the composites, spherical Ti6Al4V powders with an average particle size of 150 μm, prismatic TiB_2_ powders (3 μm) and fine Si powders (3 μm) were used. At first, Ti6Al4V, TiB_2_ and Si powders were mixed using low-energy ball milling for 5 h at 230 rpm under an argon atmosphere. The ratio of milled media to material is 5:1. Afterwards, the blended powder mixtures were hot pressed in vacuum (10^−2^) at 1300 °C for 90 min under a pressure of 20 MPa. TiBw reinforcements around the Ti6Al4V matrix were synthesized via *in situ* reaction between TiB_2_ and Ti. The reaction can be described as the equation (1)^4^. Ti_5_Si_3_ reinforcements in the β phase were achieved according to the equation (2) in the Ti-Si phase diagram^19^.


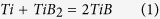



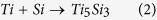


According the above processes, 4vol.%Ti_5_Si_3_/Ti6Al4V, (2vol.%Ti_5_Si_3_ + 3.4vol.%TiBw)/Ti6Al4V, (4vol.%Ti_5_Si_3_ + 3.4vol.%TiBw)/Ti6Al4V and (8vol.%Ti_5_Si_3_ + 3.4vol.%TiBw)/Ti6Al4V composites were prepared. In order to adjust the distribution of Ti_5_Si_3_ particles and investigate the contribution of the unique structure to the mechanical properties, the (4vol.%Ti_5_Si_3_ + 3.4vol.%TiBw)/Ti6Al4V composites were water quenched (WQ) after solid solution strengthening at 990 °C, 1100 °C, and 1200 °C for 40 min.

A scanning electron microscope (SEM; ZEISS Supra 55 SAPPHIRE) instrument and a transmission electron microscope (TEM; TecnaiF2F30) instrument were used to observe the microstructure of the fabricated composites. The tensile and compressive properties of the composites were carried out using an Instron-5569 universal testing device, at a crosshead speed of 0.5 mm/min. The tensile specimens were machined with the gauge size of 15 mm × 5 mm × 1.5 mm, and compressive specimens had dimensions of ø4 mm × 6 mm. At least five tests were performed on each condition and the average values were used.

The wear properties of the Ti6Al4V alloy and the fabricated composites were evaluated using a pin-on-disk tribometer (Teer POD-I). During the tests, the specimens were rotated against a stationary GCr15 steel ball of 5 mm diameter at the speed of 200 r/min for 1800 s under a contact load of 10N. The radius of the wear track was about 5 mm. The friction coefficient was continuously recorded during the tests. All tests were conducted in air (temperature 20 °C, humidity 40% RH). Before and after each wear test, the specimens were cleaned with acetone and then dried. The mass loss of the specimens was measured using an electronic balance with an accuracy of 0.01 mg. Each data point represents the average value of three test results.

## Conclusions

To sum up, novel two-scale network structured (Ti_5_Si_3_ + TiBw)/Ti6Al4V composites were successfully *in situ* fabricated using low-energy milling and reaction hot pressing. A summary of the key findings in this paper are:

1. TiBw was synthesized around the Ti6Al4V particles, and formed the first-scale network structure. Ti_5_Si_3_ precipitated in the β-Ti phase around the α-Ti phase, and formed the secondary-scale network architecture.
2. Heat treatment can adjust the size and distribution of the Ti_5_Si_3_ reinforcement. The ratio of the TiBw and Ti_5_Si_3_ reinforcements distributed in different scales can be controlled by adjusting the proportions of TiB_2_ and Si raw materials.
3. Ti_5_Si_3_ phase precipitated in the β-Ti phase has certain crystal relationships with the β-Ti phase: 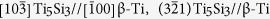.
4. The composites with two-scale network architecture exhibit superior mechanical properties over the composites with one-scale architecture and the Ti6Al4V alloy.

## Additional Information

**How to cite this article**: Jiao, Y. *et al*. Controllable two-scale network architecture and enhanced mechanical properties of (Ti_5_Si_3_+TiBw)/Ti6Al4V composites. *Sci. Rep.* **6**, 32991; doi: 10.1038/srep32991 (2016).

## Figures and Tables

**Table 1:** Tensile properties of the (4vol.%Ti_5_Si_3_ + 3.4vol.%TiBw)/Ti6Al4V composites before and after heat treatments at room temperature.

Materials	Heat treatment	σ_0.2_	σ_b_	δ_5_ (%)
1	—	1004 ± 10.4	1160 ± 9.8	4 ± 0.1
2	990 °C WQ	—	1010 ± 12.5	—
3	1100 °C WQ	—	1050 ± 11.2	—
4	1200 °C WQ	—	1070 ± 13	—

**Table 2:** Compressive properties of the (4vol.%Ti_5_Si_3_ + 3.4vol.%TiBw)/Ti6Al4V composites before and after heat treatments at room temperature.

Materials	Heat treatment	YCS(MPa)	UCS(MPa)	ε (%)
1	—	1225 ± 9.6	1402 ± 10	21.9 ± 0.5
2	990 °C WQ	1305 ± 11.3	1412 ± 11.5	16.3 ± 0.3
3	1100 °C WQ	1535 ± 12.6	1627 ± 12	8.2 ± 0.2
4	1200 °C WQ	1687 ± 12.5	1753 ± 13	6.6 ± 0.2

**Table 3:** Mass loss and COF for the Ti6Al4V alloy, the as-sintered and heat treated (4vol.%Ti_5_Si_3_ + 3.4vol.%TiBw)/Ti6Al4V composites.

Materials	Mass loss (mg)	COF
Ti6Al4V	7.98 ± 0.01	0.298
(4vol.%Ti_5_Si_3_ + 3.4vol.%TiBw)/Ti6Al4V	6.65 ± 0.04	0.277
990 °C WQ	7.71 ± 0.02	0.286
1100 °C WQ	6.73 ± 0.02	0.266
1200 °C WQ	6.30 ± 0.03	0.280
